# Circulating miR-145-5p and miR-133a-3p in pediatric sickle cell disease: biomarker potential for vaso-occlusive crises and disease activity

**DOI:** 10.1007/s00277-025-06696-1

**Published:** 2025-11-07

**Authors:** Reham Fares, Salwa Bakr, Rehab Galal Abd El-Hamid, Omayma O. Abdelaleem, Mai Mahmoud Sharabi, Mostafa Yehia Abdelwahed, Hadeel Sherbieny, Marwa Kamal, Eman Yousief Ali

**Affiliations:** 1 Department of Medical Biochemistry and Molecular Biology, Faculty of Medicine, Fayoum University, Fayoum, Egypt; 2 Department of Clinical Pathology/Hematology, Faculty of Medicine, Fayoum University, P.O. Box: 63514, Fayoum, Egypt; 3 Department of Pediatrics, Faculty of Medicine, Fayoum University, Fayoum, Egypt; 4 Department of Physiology, Faculty of Medicine, Fayoum University, Fayoum, Egypt; 5 Department of Medical Physiology, Faculty of Medicine, Zagazig University, Zagazig, Egypt; 6 Department of Clinical Pharmacy, Faculty of Pharmacy, Fayoum university, Fayoum, Egypt

**Keywords:** MicroRNAs, MiR-145, MiR-133a, Vaso-occlusive crisis, Sickle cell disease, Biomarker.

## Abstract

MicroRNAs (miRNAs) involving miR-145-5p and miR-133a-3p are increasingly recognized for their roles in modulating inflammation, oxidative stress, and vascular integrity, key processes implicated in sickle cell disease (SCD) pathophysiology. To investigate the expression levels of miR-145-5p and miR-133a-3p in pediatric SCD patients during vaso-occlusive crisis (VOC) as well as steady-state conditions, and to evaluate their potential as clinical biomarkers. A case-control study was conducted including 45 pediatric SCD patients (24 during VOC and 21 in steady-state) and 45 age- and sex-matched healthy controls. Plasma miR-145-5p and miR-133a-3p levels were quantified using real-time quantitative PCR and correlated with clinical characteristics, laboratory parameters, genotype, and hydroxyurea (HU) therapy. Circulating miR-133a-3p and miR-145-5p were significantly higher in SCD patients than in controls (p < 0.001), with a further elevation during VOC compared to steady-state (p < 0.050). While no significant association was found with genotype or HU therapy (p > 0.05), miR-133a-3p levels correlated significantly with fetal hemoglobin (HbF) (p = 0.006), reticulocyte count (p = 0.049), and urine albumin/creatinine ratio (p = 0.043). A strong positive correlation was observed between both miRNAs (r = 0.683, p < 0.001). Receiver Operating Characteristic (ROC) analysis demonstrated reasonable diagnostic accuracy of both miRNAs in distinguishing VOC from steady-state (AUC = 0.69). This study highlights the potential of miR-133a-3p and miR-145-5p as biomarkers for SCD activity. The association of miR-133a-3p with HbF and urine A/C ratio further suggests clinical relevance in vaso-occlusive complications. Larger longitudinal and functional studies are needed to validate these findings.

**Supplementary Information:**

The online version contains supplementary material available at 10.1007/s00277-025-06696-1.

## Introduction

Sickle cell disease (SCD) comprises a group of inherited hemoglobinopathies characterized by the presence of hemoglobin S (HbS), which results from a point mutation in the β-globin gene. The most common and severe form is homozygous HbSS (sickle cell anemia), while other clinically significant genotypes include compound heterozygous states such as HbSC, HbS/β⁰-thalassemia, and HbS/β⁺-thalassemia. In all forms, the presence of HbS predisposes red blood cells to polymerization under deoxygenated conditions, leading to sickling, impaired deformability, and chronic hemolytic anemia. These abnormal erythrocytes promote vaso-occlusion, chronic inflammation, and progressive multi-organ damage [1].

Vaso-occlusive crisis (VOC), the hallmark and most debilitating clinical manifestation of SCD, arises from a complex interplay of mechanisms. These include both intravascular and extravascular hemolysis, oxidative stress, abnormal cellular adhesion, endothelial dysfunction, vascular inflammation, and the activation of coagulation and complement pathways [1]. Collectively, these processes drive vaso-occlusion, tissue ischemia, establishing a self-perpetuating cycle of vascular injury through enhanced generation of reactive oxygen species (ROS) [2].

MicroRNAs (miRNAs) are short non-coding RNAs that regulate gene expression post-transcriptionally and play critical roles in diverse physiological and pathological processes [3]. They are involved in inflammation, immune regulation, oxidative stress responses, and apoptosis, as well as various disease mechanisms [4–6]. Among them, miR-133a-3p and miR-145-5p have been reported to modulate endothelial function, vascular remodeling, redox balance, and inflammatory pathways across different disease contexts [7–14]. These biological processes are central to the pathophysiology of SCD, particularly in relation to VOC [1]. However, the specific contribution and clinical relevance of these miRNAs in SCD remain insufficiently explored.

MiR-145-5p is enriched in vascular tissues and regulates endothelial homeostasis, as well as vascular smooth muscle cell differentiation and proliferation [8]. Conversely, miR-133a-3p is a muscle-specific miRNA involved in angiogenesis, fibrosis, cardiac remodeling, and apoptosis [10]. Earlier studies have shown that miR-145-5p suppresses inflammatory pathways, such as IL6 and TNF-α [7], while miR-133a protects against oxidative stress-induced apoptosis [12]. Notably, circulating miR-133a is elevated in critical illness and sepsis [5, 13], conditions that share key pathological features with SCD, including systemic inflammation, oxidative stress, endothelial dysfunction, and multi-organ injury, suggesting a potential role as a biomarker in SCD.

The present study therefore aimed to investigate the expression levels of miR-145-5p and miR-133a-3p in pediatric SCD patients during both steady-state and VOC compared to healthy controls, and to explore their associations with clinical and laboratory parameters to assess their potential as biomarkers of disease activity and severity.

## Patients and methods

### Research design and clinical setting

This case-control study was conducted between August 2024 and January 2025 at the Hematology and Pediatric Outpatient Clinics of Fayoum University Hospital. The study was performed in accordance with the ethical principles of the Declaration of Helsinki and its subsequent amendments. Ethical approval was granted by the Institutional Review Board (IRB approval no. R725), and written informed consent was secured from the legal guardians of all enrolled participants.

### Study population

The study included a total of 90 pediatric participants divided into three groups. Group I (VOC group): 24 pediatric patients with confirmed SCD who were hospitalized at the onset of VOC, clinically diagnosed by acute sever pain requiring medical attention. Group II (Steady-state group): 21 age- and sex-matched pediatric patients with confirmed SCD in steady-state, defined as no pain crisis, acute illness, or hospitalization for at least 4 weeks prior to enrollment. Group III (Control group): 45 age- and sex-matched apparently healthy children recruited from children attending the same hospital for routine check-ups or vaccination.

The sample size was calculated using G*Power software (version 3.0.10). Based on an assumed power of 90%, an alpha level of 0.05, and a large effect size (0.8) for differences in biomarker levels between SCD patients group and control group, the required sample size was estimated as 34 per group. To account for potential non-response and missing data, this was increased by 15% in 40 participants per group.

#### Inclusion Criteria

Confirmed diagnosis of SCD by hemoglobin electrophoresis or HPLC, age less than 18 years. For VOC group: Clinical diagnosis of VOC requiring hospitalization. For steady-state group: No pain crises, recent transfusion, or acute illness for at least 4 weeks.

#### Exclusion criteria

Current co-existing chronic inflammatory or autoimmune diseases (based on history, examination, and laboratory records). Recent transfusion within the preceding 4 weeks, current use of corticosteroids or immunosuppressive therapy, acute infection at the time of enrollment.

All SCD patients underwent detailed clinical evaluation, including VOC frequency, transfusion history, hydroxyurea (HU) therapy, and family history. Clinical severity of SCD was assessed using the Cameron severity score (1983), validated for pediatric by van den Tweel et al. (2010) [15, 16]. The score incorporate age at first SCD manifestation, annual hospitalizations, VOC frequency and type, pneumococcal infections, organ involvement, and growing parameters. Scores 0–4 indicate mild SCD, 5–8 moderate, and 9–21 severe (Supplementary, Table S1).

## Laboratory, and radiological assessment

All SCD participants underwent comprehensive laboratory and radiological evaluations. Laboratory tests included complete blood count (CBC) with reticulocyte parameters (Sysmex CX-1000, Sysmex Corp., Kobe, Japan), hemoglobin electrophoresis by capillary isoelectric focusing (Capillaris 2^®^ SEBIA, France), and biochemical assays including lactate dehydrogenase (LDH), bilirubin, liver and renal function using the Cobas 6000 analyzer (Roche Diagnostics, Mannheim, Germany). The urine albumin/creatinine (u A/C) ratio was measured by immunoassay (DCA Bayer analyzer^®^, Siemens Healthcare Diagnostics Pyt Ltd., Australia).

Radiological evaluation included echocardiography, abdominal ultrasound and transcranial Doppler (TCD) ultrasonography using Philips HD11 XE system. Cerebral blood flow velocities were assessed with a 2-MHz probe and interpreted according to STOP trial criteria (normal < 170 cm/s; conditional 170–199 cm/s; abnormal ≥ 200 cm/s).

Genotypes of all SCD patients were confirmed using high-performance liquid chromatography (HPLC) (Bio-Rad Variant II, Hercules, CA, USA), following the manufacturer’s protocol.

Controls underwent history taking and laboratory evaluation, including CBC, peripheral blood smear examination, sickling test and hemoglobin electrophoresis/HPLC were performed to exclude carrier status or hematological abnormalities.

## Molecular studies of MiR-133a-3p and MiR-145-5p expression levels

### Sample collection and RNA extraction

Peripheral venous blood samples (2 mL) were collected into EDTA-containing tubes. Plasma was separated within 30 min of collection by centrifugation and subsequently stored at − 80 °C until further processing.

Total RNA, including miRNAs, was isolated from 200 µL of plasma using the miRNeasy Serum/Plasma Kit (Qiagen, Germany), according to the manufacturer’s instructions. RNA concentration and purity were assessed using a NanoDrop spectrophotometer (NanoDrop Technologies, Inc., Wilmington, USA).

### Quantification of MiR-145-5p and MiR-133a-3p expression by RT-qPCR

Complementary DNA (cDNA) synthesis was performed using the miRCURY LNA RT Kit (Qiagen, Germany). Quantitative real-time PCR (RT-qPCR) was then carried out using the miRCURY LNA miRNA PCR Assays and miRCURYLNA SYBR Green Master Mix (Qiagen, Germany). For normalization, miR-16-5p small nuclear RNA was used as an endogenous control [17]. Relative expression levels were calculated using the 2^−ΔΔCt method.

## Statistical analysis

Data were analyzed using the Statistical Package for Social Science (SPSS) software, version 25.0 (IBM Corp., Armonk, NY, USA). Categorical variables were expressed as frequencies and percentages, while continuous variables were presented as mean ± standard deviation (SD) or median (interquartile range, IQR).

Comparisons between study groups were conducted using Student’s t-test, Mann–Whitney U test, or one-way ANOVA, as appropriate. The Chi-square test was used for categorical variables. Spearman correlation coefficient was applied to assess relationships between miRNA expression levels and clinical or laboratory parameters.

Receiver Operating Characteristic (ROC) curves were plotted in SPSS to assess the diagnostic performance of the studied miRNAs. A p-value < 0.05 was considered statistically significant.

## Results

This study enrolled 45 patients with SCD, including 24 during VOC (53.3%) and 21 during steady-state (46.7%), with a mean age of 9.8 ± 2.89 years. An additional 45 age- and sex-matched healthy individuals were recruited as controls. Demographic and clinical characteristics are summarized in Table 1, 2; Supplementary Table S2, S3.

**Table 1:** Demographic and Clinical Data of Study SCD Patients

**Variables**		**No. (45)**	**%**
Gender	Female	23	51.1%
	Male	22	48.9%
Presenting clinical status	Crisis (VOC)	24	53.3%
	Steady State	21	46.7%
Genotype	SB0	10	22.2%
	SB+	15	33.3%
	SS	20	44.4%
Frequency of VOC in last year	<3	14	31.1%
	3–10	28	62.2%
	>10	3	6.7%
SCD Clinical Severity Score [15, 16]	Mild	19	4>2.2%
	Moderate	17	37.8%
	Sever	9	20.0%
Sequestration crisis	No	43	95.6%
	Yes	2	4.4%
Hyper-hemolytic crisis	No	32	71.1%
	Yes	13	28.9%
Frequency of transfusion per life	<10	24	53.3%
	10–20	14	31.1%
	>20	7	15.6%
Frequency of transfusion in last year	<3	34	75.6%
	3–10	10	22.2%
	>10	1	2.2%
Acute chest syndrome (ACS)	No	34	75.6%
	Yes	11	24.4%
Priapism	No	44	97.8%
	Yes	1	2.2%
Stroke	No	43	95.6%
	Yes	2	4.4%
A vascular necrosis	No	44	97.8%
	Yes	1	2.2%
Splenomegaly	Yes	9	20.0%
	No	30	66.7%
	Splenectomy	6	13.3%
Stunted growth	No	41	91.1%
	Yes	4	8.9%
Gall stones	No	38	84.4%
	Yes	7	15.6%
Hydroxyurea (HU)	No	13	28.9%
	Yes	32	71.1%
Compliance with HU	No	13	40.6%
	Yes	19	59.4%
Response to HU	Good	18	56.3%
	Poor	14	43.7%
Chelation Therapy	Yes (Deferosirex)	11	24.4%
	No	34	75.6%
Long-Acting Penicillin	No	27	60.0%
	Yes	18	40.0%
Vaccination	No	11	24.4%
	Yes	34	75.6%

**Table 2:** Laboratory and Radiological Findings of Study SCD Patients

**Laboratory Variables**			**No. (45)**	**%**	
Genotype	SB^0^		10	22.2%	
	SB^+^		15	33.3%	
	SS		20	44.4%	
	**Mean**	**SD**	**Median**	**IQR**	
Hb F *(%)*	19.1	10.3	18.0	11.9	22.0
Hb S *(%)*	68.6	11.9	72.0	60.0	77.0
Hb A *(%)*	6.3	10.7	0.0	0.0	9.0
Hb A2 *(%)*	4.7	4.7	3.9	3.4	5.0
Hb *(g/dl)*	8.3	1.3	8.5	7.5	9.5
MCV *(fl)*	73.6	9.6	72.0	67.0	82.0
MCH	28.7	4.1	29.0	25.0	32.0
PLT *(x10*^*9*^*/L)*	437.6	152.5	433.0	344.0	556.0
TLC *(x10*^*9*^*/L)*	8.1	2.9	8.5	5.6	9.8
Retics *(%)*	2.5	0.8	2.2	1.9	2.9
Total bilirubin	2.4	0.7	2.4	1.9	2.7
Direct bilirubin	0.3	0.3	0.3	0.2	0.3
Creatinine *(mg/dl)*	0.5	0.2	0.4	0.3	0.6
ALT *(IU/L)*	41.2	16.2	37.0	32.0	44.0
AST *(IU/L)*	40.3	17.0	35.0	27.0	45.0
Ferritin *(ug/L)*	595.4	467.4	430.0	280.0	743.0
Urine A/C ratio*(mg/g)	55.1	91.6	28.0	22.0	40.0
Radiological Variables			**No. (45)**	**%**	
Trans Cranial Doppler**	Abnormal		2	4.4%	
	Conditional		5	11.1%	
	Normal		38	84.4%	
	**Mean**	**SD**	**Median**	**IQR**	**Mean**
LVIDD	3.6	0.4	3.6	3.2	4.1
LVIDS	2.4	0.5	2.4	1.9	2.8
LA diameter	2.1	0.2	2.1	1.9	2.3
ESPAP	32.5	4.3	32.0	29.0	35.0
TRV	2.3	0.2	2.3	2.2	2.4

Hb:hemoglobin

PLT: platelet cunt

TLC: total leucocytic count

LVIDD: left ventricular internal diameter in diastole

LVIDS: left ventricular internal diameter in systole

LA: left atrial

ESPAP: estimated systolic pulmonary artery pressure

TRV: Tricuspid Regurgitation Velocity

*A high uACR may be an early sign of kidney disease

**Trans Cranial Doppler results are classified into three statuses based on time-averaged mean of the maximum (TAMM) velocity in large intracranial arteries (normal <170, conditional 170–199, Abnormal 200 cm/s)

The genotypic distribution of SCD cohort was HbSS (44.4%), HbS/β⁰-thalassemia (22.2%), and HbS/β⁺-thalassemia (33.3%) (Table 1.). Both VOC and steady-state groups contained individuals from each genotype category, with no significant differences in genotype distribution between disease activity status (*P*-value of 0.159; Supplementary Table S2).

Stratification by genotype showed HbSS patients had the highest frequency of 3–10 VOC episodes/year (65%) and a greater proportion classified as mild to moderate severity (45% − 40% respectively). In contrast, HbSβ⁺ patients demonstrated a wider severity spectrum, with nearly half classified as mild (46.7%), and smaller proportions as moderate (26.7%) or sever (26.7%) (Supplementary Table S3).

Overall, miR-133a-3p and miR-145-5p expression levels were significantly higher in SCD patients compared to controls (*p* < 0.001, Table 3), and both miRNAs were elevated during VOC relative to the steady-state (*p* < 0.050, Table 4). No significant associations were observed with genotype, VOC frequency, transfusion frequency, or HU response (*p* > 0.05, Supplementary Table S4).

**Table 3:** Comparison of miR-133a-3P and miR-145-5P Expression Levels among Study Population (SCD Patients versus Control Individuals)

	**miR-133a−3P**			**miR-145-5p**		
	**Median**	**IQR**		**Median**	**IQR**	
Cases	12.13	1.41	33	6.23	3.12	17.63
Controls	1	1	1	1	1	1
P-value^#^	<0.001*			<0.001*

**Table 4:** Comparison of miR-133a-3P and miR-145-5P Expression Levels During VOC and Steady-State of Study SCD patients

	**miR-133a−3P**			**miR-145-5p**		
	**Median**	**IQR**		**Median**	**IQR**	
Crisis	18.42	9.19	33	9.54	5.83	18.94
Steady-state	1.87	0.19	24.25	3.84	2.53	7.16
P-value^#^	0.028*			0.034*		

Subgroup analysis demonstrated that miR-133a-3p expression varied across clinical severity categories (*p* = 0.014), peaking in the moderate severity group, whereas lower levels were noted in mild and severe groups (Supplementary Table S4). When severity was analyzed as a continuous score, a weak non significant correlation was (*r* = 0.256, *p* = 0.090) (Supplementary Table S5).

**Fig. 1:**
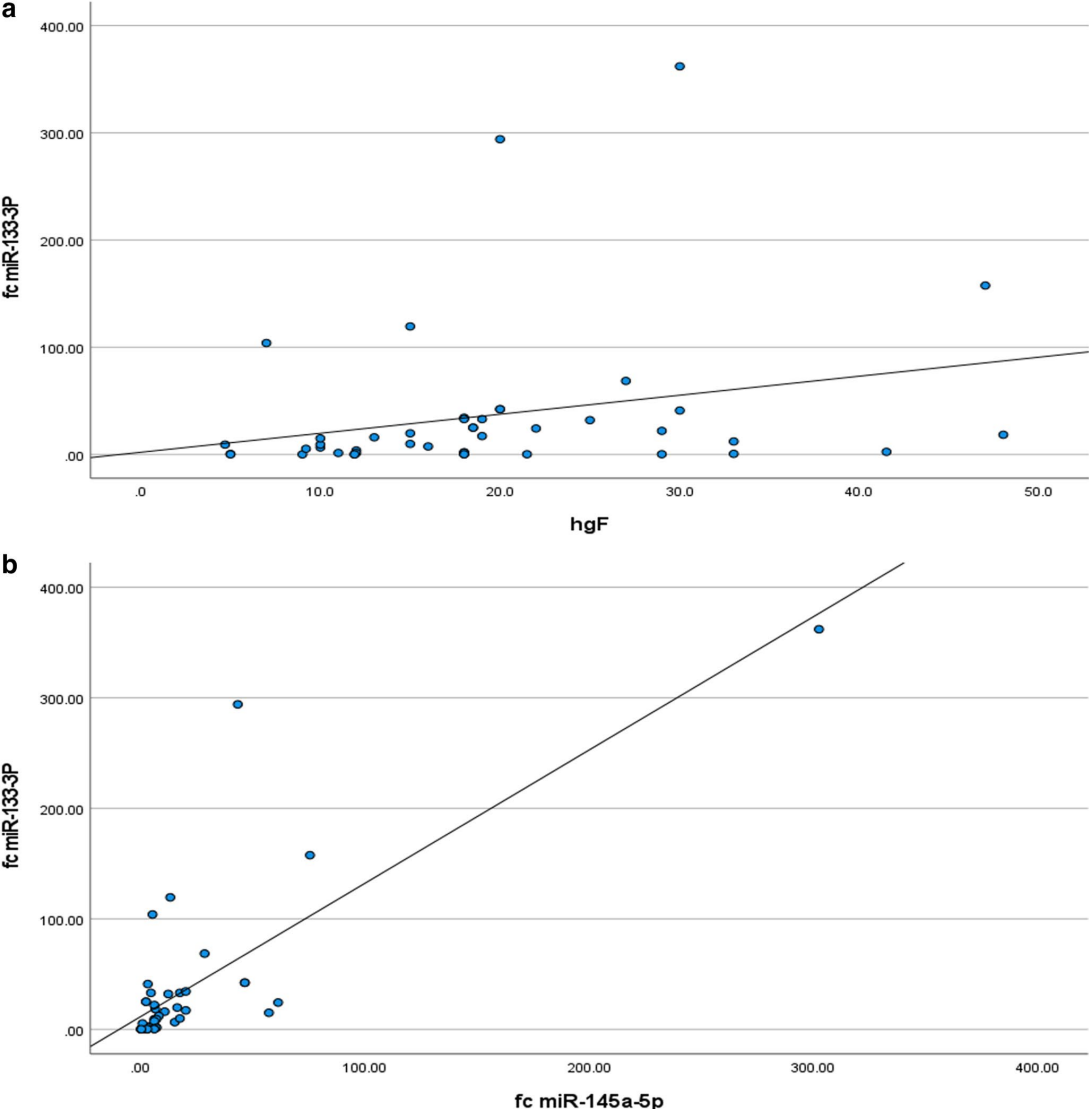
(**a**) Correlation between miR-133a-3p Expression and Fetal Hemoglobin (HbF) Levels in Study Patients. (**b**) Correlation between miR-133a-3p and miR-145-5p Expression Levels in Study SCD Patients

Interestingly, miR-133a-3p expression correlated significantly with HbF (*p* = 0.006, Fig. 1-a), reticulocyte count (*p* = 0.049), and urine A/C ratio (*p* = 0.043), but not with other laboratory or radiological parameters (*p* > 0.05, Supplementary Table S5).

Regarding HU therapy, 32 of 45 patients (71.1%) were receiving treatment, of whom 56.3% showed an excellent hematological and clinical response, while 43.7% had partial or poor responses (Table 1.). Although HU response did not differ by disease activity status (*p* > 0.05, Supplementary Table S2), it varied significantly by genotype (*p* = 0.027), being highest in HbSS (84.6%) and lowest in HbS/β⁺ patients. Importantly, miR-133a-3p and miR-145-5p expression levels did not differ between HU responders and non-responders (*p* = 0.82).

Importantly, a strong positive correlation was observed between miR-145-5p and miR-133a-3p expression levels (*r* = 0.683, *p* < 0.001, Fig. 1-b; Supplementary Table S5).

**Fig. 2:**
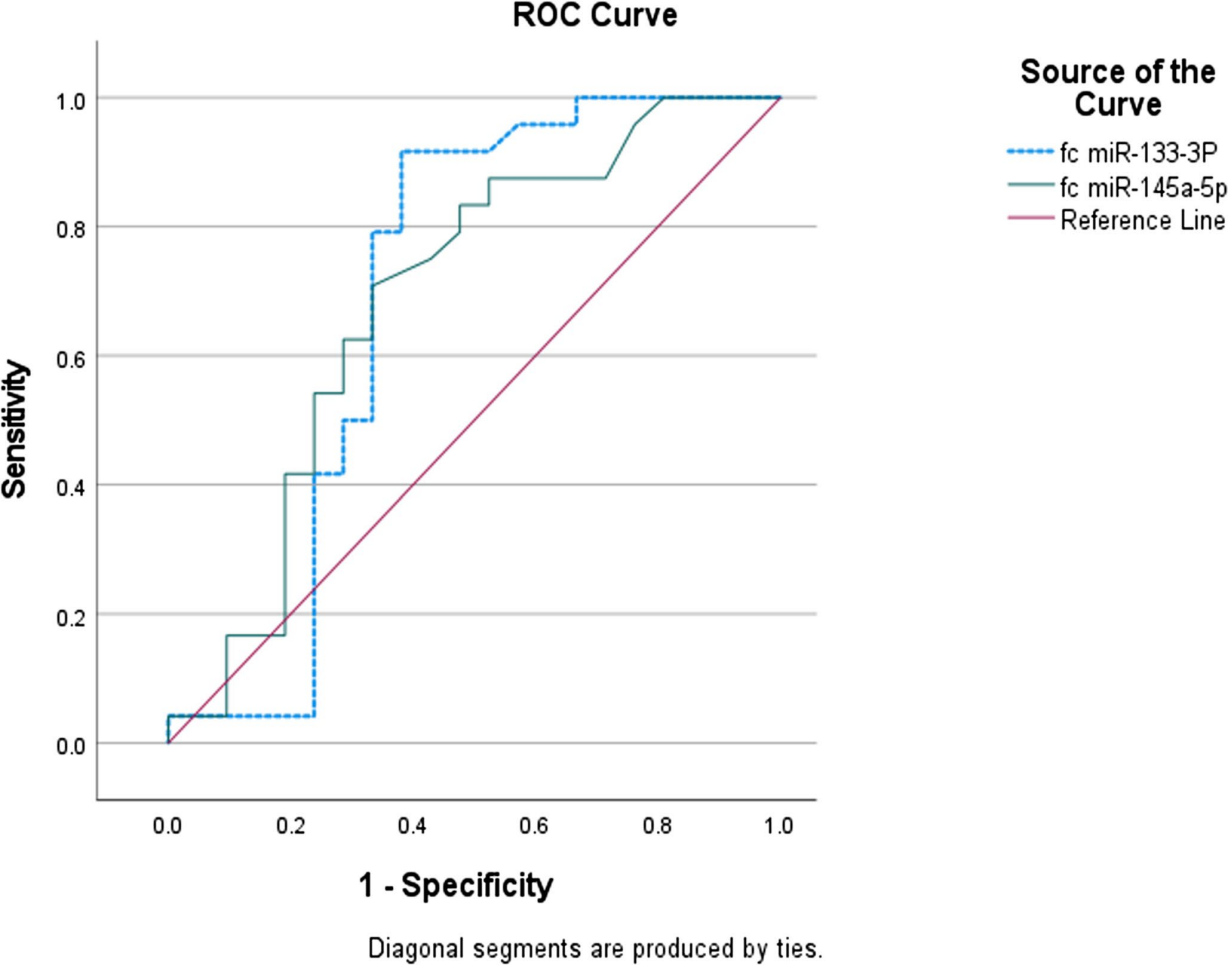
ROC curve for miR-133a-3p and miR-145-5p Expression in Differentiating Clinical Characteristics of SCD Patients (VOC versus Steady-State)

ROC curve analysis revealed that both miRNAs discriminated SCD patients from controls (AUC = 0.78 for miR-133a-3p; AUC = 0.87 for miR-145-5p; *p* < 0.001, Table 3**)** and distinguished VOC from steady-state SCD patients with moderate diagnostic accuracy (AUC = 0.69 for each; *p* < 0.05, Table 5; Fig. 2).

**Table 5:** Validity of miR-133a-3p and miR-145-5p Expression Levels in Differentiating Study SCD Patients with VOC from Steady-status

	**AUC(95% CI)**	**P-value**	**Cut-off point**	**Sensitivity (%)**	**Specificity (%)**
**ROC Curve for Differentiating Patients with SCD from Controls**					
miR-133-3P	0.778 (0.656–0.899.656.899)	<0.001*	1.207	77.8	100
miR-145a-5p	0.867 (0.767–0.966.767.966)	<0.001*	1.766	86.7	100
**ROC Curve for Differentiating SCD Patients with VOC from Steady-status**					
miR-133-3P	0.691 (0.515–0.868.515.868)	0.028*	3.097	91.7	61.9
miR-145a-5p	0.688 (0.526–0.851.526.851)	0.031*	6.233	70.8	66.7

## Discussion

Dysregulation of miR-133a-3p and miR-145-5p may contribute to the pro-inflammatory and pro-oxidant milieu of SCD, which drives VOC and organ injury. Their expression profiles provide insights into disease activity and severity, supporting their potential use as biomarkers and therapeutic targets. In this study, both miRNAs were significantly upregulated in SCD compared with healthy controls, with further elevation during VOC and across severity categories. These findings align with reports implicating both miRNAs in inflammation, oxidative stress, and vascular dysfunction, central mechanisms in SCD complications [5, 7, 8, 10–12].

Notably, miR-133a-3p expression correlated with Hb F levels, reticulocyte counts, and urine albumin/creatinine ratio, linking it to hemolysis, erythropoietic stress, and early renal involvement. These associations suggest a potential role in modulating vaso-occlusive complications and underscore its therapeutic relevance. Although miR-145-5p showed no associations with specific clinical or laboratory parameters, its consistent overexpression in SCD, particularly during VOC, supports its role as a systemic marker of endothelial and inflammatory stress. The strong correlation between the two miRNAs further indicates they may act in shared regulatory pathways.

The elevated expression of both miRNAs during VOC suggests a possible compensatory upregulation in response to inflammatory and vascular stress. Prior studies support this notion, miR-133a-3p regulates oxidative stress pathways, enhances antioxidant activity, and suppresses ROS [18], while miR-145-5p modulate vascular integrity and cytokine release [19]. Together, they may represent a synergistic response to endothelial injury.

Our results also showed higher miR-133a-3p expression in moderate disease severity, but no linear correlation with continuous severity scores. This may reflect a non-linear or U-shaped relationship: moderate disease may drive peak miRNA upregulation through active inflammatory signaling, whereas mild disease lacks sufficient stimulus and severe disease may exhibit exhaustion or regulatory suppression. This interpretation is consistent with the limited number of severe cases in our cohort.

ROC analysis demonstrated strong diagnostic accuracy of both miRNAs for distinguishing SCD patients from controls (AUC = 0.78 for miR-133a-3p; AUC = 0.87 for miR-145-5p), supporting their potential as non-invasive biomarkers. In contrast, their ability to differentiate VOC from steady state was modest (AUC = 0.69 for both), likely reflecting overlapping biological pathways and the modifying effects of hydroxyurea (HU). Although HU response did not differ by disease activity status (*p* > 0.05), it varied significantly by genotype (*p* = 0.027), being highest in HbSS (84.6%) and lowest in HbS/β⁺ patients (33.3%). Importantly, miR-133a-3p and miR-145-5p expression levels did not differ significantly between HU responders and non-responders, suggesting that HU therapy may not directly confound their biomarker potential.

Beyond SCD, accumulating evidence supports roles for these miRNAs in diverse pathologies. MiR-133a-3p regulates angiogenesis, apoptosis, oxidative stress, and fibrosis [10–12, 20–23], while its serum levels correlate with sepsis severity and outcomes [5, 13]. In cardiovascular and fibrotic disorders, it modulates endothelial dysfunction, ROS accumulation, and remodeling pathways. Similarly, miR-145-5p, well established as a tumor suppressor, has emerging roles in vascular inflammation via NF-κB activation, cytokine release, and PI3K/AKT signaling [3, 7–9]. Its functions extend to angiogenesis, vascular remodeling, and tissue repair [3, 9, 14].

Together, these findings suggest that miR-133a-3p and miR-145-5p may serve not only as biomarkers of SCD activity but also active contributors to disease pathogenesis and therapeutic targets for modulating oxidative and vascular pathways. However, the case-control design and the modest, single-center sample size may limit causal inference and generalizability. Larger longitudinal and functional studies are warranted to validate their regulatory roles and therapeutic potential.

## Conclusion

This study highlights the potential of miR-133a-3p and miR-145-5p as biomarkers of SCD activity. While unrelated to genotype or HU therapy, the association of miR-133a-3p with HbF and urine A/C ratio further suggests a potential modulatory role in vaso-occlusive complications. Further validation in larger longitudinal cohorts and functional studies to validate target genes and signaling pathways modulated by miR-133a-3p and miR-145-5p are warranted.

## Supplementary Information

Below is the link to the electronic supplementary material.

Supplementary Material 1(DOCX 43.5 KB)

## Data Availability

No datasets were generated or analysed during the current study.
