# Supplementary material for: Circulating miR-145-5p and miR-133a-3p in pediatric sickle cell disease: biomarker potential for vaso-occlusive crises and disease activity
Source: Ann Hematol. 2025 Nov 7;104(11):5625–33. doi: 10.1007/s00277-025-06696-1 (PMC12672820; doi:10.1007/s00277-025-06696-1)
Supplement: Supplementary file 1 — Supplementary Material 1(DOCX 43.5 KB) [file 277_2025_6696_MOESM1_ESM.docx]

**Table (S1): Clinical Classification Criteria of Severity in Sickle Cell Disease:**

|  | **Points** |
| --- | --- |
| 1. **Age at diagnosis**   <12 months  12-24 months  25 months up to 5 years  >5 years | 3  2  1  0 |
| 1. **Number of hospitalizations**   ≥ 5  2-4  1 | 3  2  1 |
| 1. **Crisis**   Sequestration  Aplastic  Thrombotic (requiring hospitalization) | 3  2  1 |
| 1. **Pneumococcal Sepsis** | 3 |
| 1. **Major organ involvement**   Cerebrovascular accident, pulmonary infarct, or retinopathy.  Pneumonia, renal or bone involvement, priapism, leg ulcer, or CHF. | 3  2 |
| 1. **Failure to thrive**   <3 percentile, height and weight  <10 percentile  <25 percentile | 3  2  1 |

**(Cameron et al., 1983)**

**Table (S2) Disease Status Distribution for Clinical Characteristics of SCD Patients:**

| Clinical Characteristics | | VOC  (n=24)  no. (%) | | Steady-state  (n=21)  no. (%) | | P-value |
| --- | --- | --- | --- | --- | --- | --- |
| Genotype | **HbSS** (n=20) | 9 (45.0) | | 11 (55.0) | | 0.159 |
|  | **HbSβ⁰** (n=10) | 8 (80.0) | | 2 (20.0) | |  |
|  | **HbSβ⁺** (n=15) | 7 (46.7) | | 8 (53.3) | |  |
| Severity Score | **Mild≤4** (n=19) | 4 (16.7) | | 15 (71.4) | | 0.001* |
|  | **Moderate (5-8)** (n=17) | 13 (54.2) | | 4 (19.0) | |  |
|  | **Sever≥9** (n=9) | 7 (29.2) | | 2 (9.5) | |  |
| VOC Frequency | **<3** (n=14) | 3 (12.5) | | 11 (52.4) | | 0.008* |
|  | **3-10** (n=28) | 18 (75.0) | | 10 (47.6) | |  |
|  | **>10** (n=3) | 3 (12.5) | | 0 (0.0) | |  |
| Clinical Events | **Sequestration crisis** (n=2) | 2 (8.3) | | 0 (0.0) | | 0.491 |
|  | **Hyper-hemolytic crisis** (n=13) | 11 (45.8) | | 2 (9.5) | | 0.007* |
|  | **Acute chest syndrome** (n=11) | 9 (37.5) | | 2 (9.5) | | 0.029* |
|  | **Priapism** (n=1) | 1 (4.2) | | 0 (0.0) | | 1.000 |
|  | **Stroke** (n=2) | 2 (8.3) | | 0 (0.0) | | 0.491 |
|  | **A vascular necrosis** (n=1) | 1 (4.2) | | 0 (0.0) | | 1.000 |
|  | **Splenomegaly** (n=9) | 3 (12.5) | | 6 (28.6) | | 0.033* |
|  | **Stunted growth** (n=4) | 3 (12.5) | | 1 (4.8) | | 0.611 |
|  | **Gall stones** (n=7) | 5 (20.8) | | 2 (9.5) | | 0.422 |
| Vaccination Status (n-45) | **No** | 3 (12.5) | | 8 (38.1) | | 0.046* |
|  | **Yes** | 21 (87.5) | | 13 (61.9) | |  |
| Response to HU  (n-32) | **Good** (n=18) | 11 (52.4) | | 7 (63.6) | | 0.819 |
|  | **Poor/ no** (n=14) | 10 (47.6) | | 4 (36.4) | |  |
| Trans Cranial Doppler | **Abnormal** | 2 (8.3) | | 0 (0.0) | | 0.027* |
|  | **Conditional** | 5 (20.8) | | 0 (0.0) | |  |
|  | **Normal** | 17 (70.8) | | 21 (100.0) | |  |
| Laboratory Findings | **Hb F** *(%)* | 18.2 | 7.7 | 20.2 | 12.7 | 0.534 |
|  | **Hb S** *(%)* | 69.1 | 10.1 | 68.0 | 13.9 | 0.764 |
|  | **Hb A** *(%)* | 6.2 | 11.1 | 6.4 | 10.6 | 0.946 |
|  | **Hb A2** *(%)* | 5.8 | 6.2 | 3.6 | 0.9 | 0.113 |
|  | **Hb** *(g/dl)* | 8.2 | 1.4 | 8.5 | 1.2 | 0.531 |
|  | **MCV** *(fl)* | 69.9 | 9.7 | 77.8 | 7.8 | 0.005* |
|  | **MCH** | 27.7 | 4.0 | 30.0 | 4.1 | 0.064 |
|  | **PLT** *(x10^9^/L)* | 475.6 | 155.1 | 394.1 | 140.6 | 0.073 |
|  | **TLC** *(x10^9^/L)* | 7.4 | 2.8 | 8.9 | 3.0 | 0.086 |
|  | **Retics** *(%)* | 2.7 | 0.9 | 2.2 | 0.6 | 0.051 |
|  | **Creatinine** *(mg/dl)* | 0.5 | 0.2 | 0.4 | 0.2 | 0.199 |
|  | **ALT** *(IU/L)* | 47.4 | 17.4 | 34.0 | 11.2 | 0.004* |
|  | **AST** *(IU/L)* | 44.8 | 18.9 | 35.2 | 13.0 | 0.056 |
|  | **Ferritin** *(ug/L)* | 688.6 | 508.5 | 488.9 | 401.1 | 0.155 |
|  | **Urine A/C ratio*** *(mg/g)* | 55.0 | 66.5 | 55.1 | 115.7 | 0.996 |

**Table (S3) Genotype Distribution for Clinical Characteristics of SCD Patients:**

| Clinical Characteristics | | HbSS  (n=20)  no. (%) | | HbSβ⁰  (n=10)  no. (%) | | HbSβ⁺  (n=15)  no. (%) | | P-value |
| --- | --- | --- | --- | --- | --- | --- | --- | --- |
| Disease State | **VOC** (n=24) | 9 (45.0) | | 8 (80.0) | | 7 (46.7) | | 0.159 |
|  | **Steady-state** (n=21) | 11 (55.0) | | 2 (20.0) | | 8 (53.3) | |  |
| Severity  Score | **Mild≤4** (n=19) | 9 (45.0) | | 3 (30.0) | | 7 (46.7) | | 0.744 |
|  | **Moderate (5-8)** (n=17) | 8 (40.0) | | 5 (50.0) | | 4 (26.7) | |  |
|  | **Sever≥9** (n=9) | 3 (15.0) | | 2 (20.0) | | 4 (26.7) | |  |
| VOC Frequency | **<3** (n=14) | 7 (35.0) | | 0 (0.0) | | 7 (46.7) | | 0.004* |
|  | **3-10** (n=28) | 13 (65.0) | | 7 (70.0) | | 8 (53.3) | |  |
|  | **>10** (n=3) | 0 (0.0) | | 3 (30.0) | | 0 (0.0) | |  |
| Clinical  Events | **Sequestration crisis** (n=2) | 0 (0.0) | | 0 (0.0) | | 2 (13.3) | | 0.123 |
|  | **Hyper-hemolytic crisis** (n=13) | 2 (10.0) | | 6 (60.0) | | 5 (33.3) | | 0.016* |
|  | **Acute chest syndrome** (n=11) | 2 (10.0) | | 4 (40.0) | | 5 (33.3) | | 0.122 |
|  | **Priapism** (n=1) | 0 (0.0) | | 1 (10.0) | | 0 (0.0) | | 0.167 |
|  | **Stroke** (n=2) | 0 (0.0) | | 2 (20.0) | | 0 (0.0) | | 0.026* |
|  | **A vascular necrosis** (n=1) | 0 (0.0) | | 0 (0.0) | | 1 (6.7) | | 0.360 |
|  | **Splenomegaly** (n=9) | 2 (10.0) | | 0 (0.0) | | 7 (46.7) | | 0.001* |
|  | **Stunted growth** (n=4) | 1 (5.0) | | 2 (20.0) | | 1 (6.7) | | 0.370 |
|  | **Gall stones** (n=7) | 4 (20.0) | | 2 (20.0) | | 1 (6.7) | | 0.508 |
| Vaccination Status (n-45) | **No** | 6 (30.0) | | 1 (10.0) | | 4 (26.7) | | 0.471 |
|  | **Yes**** | 14 (70.0) | | 9 (90.0) | | 11 (73.3) | |  |
| Response to HU (n-32) | **Good** (n=18) | 11 (84.6) | | 4 (40.0) | | 3 (33.3) | | 0.027* |
|  | **Poor/ no** (n=14) | 2 (15.4) | | 6 (60.0) | | 6 (66.7) | |  |
| Trans Cranial Doppler | **Abnormal** | 0 (0.0) | | 2 (20.0) | | 0 (0.0) | | 0.094 |
|  | **Conditional** | 3 (15.0) | | 1 (10.0) | | 1 (6.7) | |  |
|  | **Normal** | 17 (85.0) | | 7 (70.0) | | 14 (93.3) | |  |
| Laboratory Findings | **Hb F** *(%)* | 24.8 | 12.0 | 18.5 | 3.7 | 12.1 | 5.3 | 0.001*  0.171^(a)^  <0.001^(b)*^  0.198^(c)^ |
|  | **Hb S** *(%)* | 70.0 | 11.5 | 76.5 | 4.3 | 61.5 | 12.3 | 0.004*  0.264^(a)^  0.063^(b)^  0.004^(c)*^ |
|  | **Hb A** *(%)* | 0.2 | 0.8 | 0.0 | 0.0 | 18.6 | 10.9 | <0.001*  0.997^(a)^  <0.001^(b)*^  <0.001 ^(c)*^ |
|  | **Hb A2** *(%)* | 4.8 | 7.0 | 5.2 | 1.2 | 4.3 | 0.9 | 0.881 |
|  | **Hb** *(g/dl)* | 9.0 | 1.2 | 7.8 | 1.2 | 7.8 | 1.0 | 0.005*  0.031^(a)*^  0.009^(b)*^  0.994^(c)^ |
|  | **MCV** *(fl)* | 80.9 | 6.8 | 63.7 | 5.8 | 70.4 | 7.2 | <0.001*  <0.001^(a)*^  <0.001^(b)*^  0.048^(c)*^ |
|  | **MCH** | 31.8 | 2.2 | 24.5 | 2.5 | 27.5 | 3.9 | <0.001*  <0.001^(a)*^  <0.001^(b)*^  0.046^(c)*^ |
|  | **PLT** *(x10^9^/L)* | 445.7 | 127.5 | 472.2 | 166.4 | 403.6 | 176.0 | 0.528 |
|  | **TLC** *(x10^9^/L)* | 8.7 | 3.0 | 6.7 | 2.5 | 8.2 | 3.0 | 0.229 |
|  | **Retics** *(%)* | 2.3 | 0.7 | 3.0 | 1.1 | 2.4 | 0.7 | 0.086 |
|  | **Creatinine** *(mg/dl)* | 0.5 | 0.2 | 0.5 | 0.2 | 0.4 | 0.1 | 0.057 |
|  | **ALT** *(IU/L)* | 37.0 | 9.5 | 47.6 | 26.3 | 42.5 | 14.1 | 0.226 |
|  | **AST** *(IU/L)* | 33.8 | 12.8 | 47.0 | 22.3 | 44.6 | 15.7 | 0.062 |
|  | **Ferritin** *(ug/L)* | 471.0 | 363.0 | 684.4 | 547.9 | 701.9 | 524.1 | 0.284 |
|  | **Urine A/C ratio** *(mg/g)* | 35.7 | 37.1 | 81.3 | 98.9 | 63.5 | 130.9 | 0.407 |

*Significant at p<0.050. ** all age-appropriate immunizations, including pneumococcal, meningococcal, Hib, and annual influenza vaccines. (a) HbSS vs. HbSβ⁰ (b) HbSS vs. HbSβ⁺ (c) HbSβ⁺ vs. HbSβ⁺.

**Table (S4): Relations between Clinical Characteristic with miR-133-3P and miR-145-5P:**

|  | | miR-133-3P | | | P-value | miR-145-5P | | | P-value |
| --- | --- | --- | --- | --- | --- | --- | --- | --- | --- |
|  |  | **Median** | **IQR** | |  | **Median** | **IQR** | |  |
| Consanguinity | **negative** | 18.38 | 1.87 | 32 | 0.31 | 7.16 | 6.23 | 12.47 | 0.52 |
|  | **positive** | 9.52 | 0.47 | 33 |  | 5.83 | 2.71 | 17.63 |  |
| Family History | **no** | 16 | 5.28 | 25 | 0.34 | 7.16 | 3.84 | 20.25 | 0.37 |
|  | **yes** | 9.52 | 0.2 | 33.65 |  | 6.23 | 2.91 | 17.04 |  |
| Frequency of VOC  *(Last year)* | **<3** | 4.96 | 0.47 | 19.7 | 0.09 | 6.92 | 2.71 | 13.36 | 0.10 |
|  | **3-10** | 13.56 | 1.71 | 32.5 |  | 5.83 | 2.91 | 16.49 |  |
|  | **>10** | 42.22 | 33 | 42.22 |  | 46.53 | 17.63 | 46.53 |  |
| SCD Clinical Severity Score** | **Mild** | 2.00 | 0.19 | 19.70 | **0.014^*^** | 6.23 | 2.71 | 15.35 | 0.32 |
|  | **Moderate** | 25.00 | 15.00 | 42.22 |  | 10.85 | 5.43 | 20.25 |  |
|  | **Severe** | 9.85 | 15.00 | 25.00 |  | 6.23 | 2.71 | 17.63 |  |
| Sequestration Crisis | **no** | 15 | 1.41 | 33 | 0.85 | 6.23 | 2.71 | 17.63 | 0.62 |
|  | **yes** | 9.52 | 9.19 | 9.85 |  | 11.93 | 6.23 | 17.63 |  |
| Hyperhemolytic Crisis | **no** | 6.98 | 0.34 | 25 | 0.09 | 6.23 | 2.62 | 12.91 | 0.07 |
|  | **yes** | 19.7 | 9.85 | 34.3 |  | 16.45 | 6.23 | 20.25 |  |
| Blood Transfusion/  per life | **<10** | 8.33 | 0.94 | 37.65 | 0.33 | 6.92 | 2.71 | 18.35 | 0.07 |
|  | **10-20** | 12.59 | 0.57 | 25 |  | 5.04 | 2.53 | 10.85 |  |
|  | **>20** | 33 | 9.85 | 42.22 |  | 17.63 | 5.43 | 46.53 |  |
| ACS | **no** | 8.33 | 0.47 | 25 | 0.07 | 6.23 | 2.53 | 15.35 | 0.10 |
|  | **yes** | 33 | 9.19 | 42.22 |  | 10.85 | 5.43 | 46.53 |  |
| Pallor | **no** | 10.66 | 2.46 | 22 | 0.92 | 6.46 | 6.23 | 8.22 | 0.74 |
|  | **yes** | 15 | 0.57 | 33 |  | 6.23 | 2.53 | 20.25 |  |
| Jaundice | **no** | 6.5 | 0.22 | 25 | 0.16 | 6.23 | 2.53 | 7.16 | **0.04*** |
|  | **yes** | 15.5 | 3.73 | 34.3 |  | 9.54 | 4.72 | 20.25 |  |
| Splenomegaly | **no** | 10.66 | 0.22 | 33 | 0.57 | 6.23 | 2.53 | 15.35 | 0.17 |
|  | **Splenoctomized** | 16.57 | 9.19 | 42.22 |  | 15.55 | 7.16 | 46.53 |  |
|  | **yes** | 9.85 | 5.28 | 24.25 |  | 6.23 | 3.84 | 17.63 |  |
| Stunted Growth | **no** | 9.19 | 1.41 | 25 | 0.08 | 6.23 | 2.71 | 17.63 | 0.47 |
|  | **yes** | 33 | 24.5 | 76.21 |  | 12.11 | 7.79 | 15.5 |  |
| Hydroxyurea (HU) | **no** | 2.46 | 1.41 | 18.38 | 0.07 | 6.68 | 2.71 | 7.16 | 0.54 |
|  | **yes** | 16.57 | 2.57 | 37.65 |  | 6.23 | 3.12 | 18.94 |  |
| Compliance | **not** | 22 | 0.22 | 32 | 0.19 | 6.23 | 3.12 | 10.85 | 0.39 |
|  | **yes** | 15 | 3.73 | 42.22 |  | 15.35 | 4.72 | 28.64 |  |
| Response to HU | **good** | 19.57 | 1.41 | 34.3 | 0.20 | 7.23 | 2.53 | 20.25 | 0.82 |
|  | **poor** | 15.5 | 3.73 | 41 |  | 5.83 | 3.34 | 17.63 |  |
| Chelation  Therapy | **deferosirex** | 16 | 3.73 | 42.22 | 0.37 | 10.85 | 4.72 | 46.53 | 0.40 |
|  | **no** | 9.19 | 1.41 | 25 |  | 6.23 | 2.71 | 15.35 |  |
| LAP | **no** | 7.46 | 0.19 | 34.3 | 0.37 | 6.23 | 2.53 | 16.45 | 0.22 |
|  | **yes** | 13.56 | 5.28 | 33 |  | 7.16 | 5.43 | 17.63 |  |
| Vaccination | **no** | 2.46 | 0.09 | 19.7 | 0.07 | 6.23 | 0.42 | 12.47 | 0.24 |
|  | **yes** | 15.5 | 1.87 | 41 |  | 6.7 | 3.12 | 20.25 |  |
| Gall stones | **no** | 13.56 | 1.87 | 34.3 | 0.43 | 6.92 | 3.12 | 17.63 | 0.51 |
|  | **yes** | 9.85 | 0.22 | 22 |  | 6.23 | 0.96 | 17.63 |  |
| Genotype | **SB 0** | 32.5 | 6.5 | 34.3 | 0.39 | 16.49 | 4.72 | 20.25 | 0.12 |
|  | **SB+** | 9.19 | 1.87 | 16 |  | 6.23 | 5.43 | 16.45 |  |
|  | **SS** | 15.25 | 0.4 | 54.8 |  | 4.79 | 0.96 | 10.79 |  |

*Significant at p<0.05. ** prescribed by Cameron et al. (1983)

**Table (S5): The Association between both miR-133a-3p and miR-145-5p Expression Levels and Clinical Characteristics of the SCD cohort:**

|  |  | miR-133a-3P | miR-145-5p |
| --- | --- | --- | --- |
| miR-145a-5p | r | **0.683** |  |
|  | P-value | **<0.001*** |  |
| Age | r | 0.252 | 0.12 |
|  | P-value | 0.094 | 0.431 |
| Duration of disease | r | 0.235 | 0.051 |
|  | P-value | 0.12 | 0.74 |
| Dose | r | 0.094 | 0.074 |
|  | P-value | 0.610 | 0.689 |
| Clinical Severity Score | r | 0.256 | 0.059 |
|  | P-value | 0.090 | 0.701 |
| Hb F | r | **0.400** | 0.281 |
|  | P-value | **0.006*** | 0.061 |
| Hb S | r | 0.082 | -0.139 |
|  | P-value | 0.593 | 0.364 |
| Hb A | r | -0.264 | 0.000 |
|  | P-value | 0.08 | 0.998 |
| Hb A2 | r | -0.013 | 0.288 |
|  | P-value | 0.93 | 0.055 |
| Hb | r | -0.082 | -0.155 |
|  | P-value | 0.594 | 0.31 |
| MCV | r | -0.036 | -0.273 |
|  | P-value | 0.814 | 0.069 |
| MCH | r | -0.068 | -0.174 |
|  | P-value | 0.658 | 0.253 |
| PLT | r | -0.022 | -0.003 |
|  | P-value | 0.884 | 0.983 |
| TLC | r | -0.143 | -0.102 |
|  | P-value | 0.348 | 0.503 |
| RET* | r | **0.294** | 0.268 |
|  | P-value | **0.049*** | 0.075 |
| Total billirubin | r | 0.097 | 0.054 |
|  | P-value | 0.526 | 0.723 |
| Direct billirubin | r | -0.08 | -0.208 |
|  | P-value | 0.601 | 0.17 |
| Serum Creatine | r | 0.12 | -0.078 |
|  | P-value | 0.432 | 0.611 |
| ALT | r | 0.213 | 0.034 |
|  | P-value | 0.161 | 0.825 |
| AST | r | 0.269 | 0.231 |
|  | P-value | 0.074 | 0.128 |
| Ferritin | r | 0.147 | 0.052 |
|  | P-value | 0.335 | 0.734 |
| Urine A/C ratio | r | **0.303** | 0.091 |
|  | P-value | **0.043*** | 0.554 |
| LVIDD | r | 0.1 | 0.176 |
|  | P-value | 0.515 | 0.247 |
| LVIDS | r | 0.104 | 0.047 |
|  | P-value | 0.498 | 0.757 |
| LA diameter | r | -0.003 | 0.028 |
|  | P-value | 0.984 | 0.857 |
| ESPAP | r | -0.022 | -0.027 |
|  | P-value | 0.884 | 0.858 |
| TRV | r | 0.183 | 0.203 |
|  | P-value | 0.229 | 0.181 |

* Reticulocyte count
